# Effects of cobalt precursor on pyrolyzed carbon-supported cobalt-polypyrrole as electrocatalyst toward oxygen reduction reaction

**DOI:** 10.1186/1556-276X-8-478

**Published:** 2013-11-14

**Authors:** Xianxia Yuan, Xin-Xin Hu, Xin-Long Ding, Hai-Chuan Kong, Hao-Dong Sha, He Lin, Wen Wen, Guangxia Shen, Zhi Guo, Zi-Feng Ma, Yong Yang

**Affiliations:** 1 Department of Chemical Engineering, Shanghai Jiao Tong University, Shanghai 200240, China; 2 School of Chemistry and Molecular Engineering, East China University of Science and Technology, Shanghai 200237, China; 3 Shanghai Synchrotron Radiation Facility, Shanghai Institute of Applied Physics, Chinese Academic of Science, Shanghai 201204, China; 4 Institute of Micro-Nano Science and Technology, Shanghai Jiao Tong University, Shanghai 200240, China; 5 State Key Laboratory of Physical Chemistry of Solid Surface & Department of Chemistry, Xiamen University, Xiamen 361005, China

**Keywords:** Non-precious metal electrocatalyst, Oxygen reduction reaction, Cobalt precursor, Catalytic active site

## Abstract

A series of non-precious metal electrocatalysts, namely pyrolyzed carbon-supported cobalt-polypyrrole, Co-PPy-TsOH/C, are synthesized with various cobalt precursors, including cobalt acetate, cobalt nitrate, cobalt oxalate, and cobalt chloride. The catalytic performance towards oxygen reduction reaction (ORR) is comparatively investigated with electrochemical techniques of cyclic voltammogram, rotating disk electrode and rotating ring-disk electrode. The results are analyzed and discussed employing physiochemical techniques of X-ray diffraction, transmission electron microscopy, Raman spectroscopy, X-ray photoelectron spectroscopy, inductively coupled plasma, elemental analysis, and extended X-ray absorption fine structure. It shows that the cobalt precursor plays an essential role on the synthesis process as well as microstructure and performance of the Co-PPy-TsOH/C catalysts towards ORR. Among the studied Co-PPy-TsOH/C catalysts, that prepared with cobalt acetate exhibits the best ORR performance. The crystallite/particle size of cobalt and its distribution as well as the graphitization degree of carbon in the catalyst greatly affects the catalytic performance of Co-PPy-TsOH/C towards ORR. Metallic cobalt is the main component in the active site in Co-PPy-TsOH/C for catalyzing ORR, but some other elements such as nitrogen are probably involved, too.

## Background

With advantages of high power density, low-operating temperature, and low emissions, proton exchange membrane fuel cells (PEMFCs) have become new electrical sources for transportable and stationary applications in recent years. There is no doubt that oxygen reduction reaction (ORR) in the cathode of PEMFCs is a key factor determining the cell performance. The ORR generally proceeds by two pathways [1]: the direct four-electron-transfer reduction of oxygen that produces H_2_O and the two-electron-transfer reduction of oxygen yielding H_2_O_2_ which may be further reduced to H_2_O. Between them, the former route is an ideal path. Therefore, it is imperative to find an efficient catalyst that can enhance the direct four-electron-transfer reduction of oxygen to give H_2_O, in order to improve the efficiency of PEMFCs. To date, carbon supported Pt and/or its alloys have been widely accepted to be the most active catalyst for ORR, but the high cost and limited resource of Pt greatly hinder the large-scale commercialization. Hence, the development of low cost, efficient and stable non-precious metal catalysts for ORR has become the goal of worldwide fuel cell people.

In the last few decades, several types of non-precious metal ORR catalysts, including transition metal macrocyclic compounds [2,3] and chalcogenides [4,5], enzymatic catalysts [6], inorganic oxide composites [7], and conducting polymers or nitrogen containing catalysts [8-10], have been explored and the heat-treated transition metal-based nitrogen-containing complexes [11-17], such as porphyrins, phthalocyanines, dibenzotetraazaannulenes, phenanthrolines, polypyrrole (PPy), triethylenetetramine chelate, tripyridyl triazine, have been considered to be the most promising alternate. Among them, PPy has been paid much more attention because of the porous structure, high surface area, high conductivity, easy synthesis and excellent environmental adaptability [18,19]. It can be used as a carrier of transition metal in the nitrogen-containing complex catalysts, where the metal particles can be fixed on its surface and physically dispersed, the interaction between PPy and metal particles can work as efficient active site for ORR [20,21]. Recent researches on transition metal-based PPy-containing catalyst Co-PPy/C [1,10,21,22] have demonstrated promising ORR activity and durability with both electrochemical experiments and single-cell performance measurements. More work is needed, however, to identify the ORR mechanism, the actual ORR active site and the effects of preparation techniques/parameters on the catalytic performance of this kind of catalyst.

In our previous work [10], a *p*-Toluenesulfonic acid (TsOH)-doped Co-PPy/C catalyst, namely Co-PPy-TsOH/C, has been successfully developed. The H_2_-O_2_ PEMFC with it as the cathode catalyst exhibited a peak power density of 203 mW · cm^−2^ with no back pressure used on either side of the cell. In the present research, a series of Co-PPy-TsOH/C catalysts have been synthesized with various cobalt precursors, and the catalytic performance towards ORR has been comparatively investigated in order to explore the effect of cobalt precursor. Then, diverse physiochemical techniques, such as X-ray diffraction (XRD), transmission electron microscopy (TEM), Raman spectroscopy, X-ray photoelectron spectroscopy (XPS), inductively coupled plasma (ICP), elemental analysis (EA), and extended X-ray absorption fine structure (EXAFS) analysis, have been employed to understand the results.

## Methods

### Synthesis of Co-PPy-TsOH/C catalysts

The Co-PPy-TsOH/C catalysts were synthesized from various cobalt precursors with a procedure previously reported [23]. Specifically, 0.6 g BP2000 carbon powder (Cabot company, Boston, MA, USA), previously treated with 6 M HNO_3_ for 8 h at 100°C, was ultrasonically dispersed in 100 ml isopropyl alcohol for 30 min, followed by an addition of 3 mmol of freshly distilled pyrrole and 100 ml double-distilled water and stirring for another 30 min. Subsequently, 100 ml ammonium peroxydisulfate solution with a concentration of 0.06 M and 0.1902 g TsOH were added and then stirred at room temperature for 4 h. Finally, the mixture was filtered, washed at least 3 times with double distilled water and alcohol alternately, and then dried at 45°C under vacuum for 12 h to obtain PPy-modified carbon which is named as PPy-TsOH/C. Then, 0.5 g PPy-TsOH/C and appropriate amount of cobalt salt (cobalt chloride, cobalt nitrate, cobalt oxalate, or cobalt acetate) were blended with 200 ml double-distilled water. After ultrasonic mixing for 1 h and vigorous stirring for 2 h, the solvent was evaporated under reduced pressure. The obtained powders were then heat-treated at 800°C for 2 h under an argon atmosphere to obtain the Co-PPy-TsOH/C catalysts.

In all the prepared catalysts, the content of Co was designed to be about 10.55% according to Equation 1, where *M* is the molecular weight of cobalt precursor, *m* is the weight of the precursor, *n* is the number of Co atom in the precursor molecule, 59 is atomic weight of cobalt, and 0.5 is the weight of PPy-TsOH/C.

(1) $$\frac{m\times \frac{n\times 59}{M}}{0.5+m\times \frac{n\times 59}{M}}\times 100\%=10.55\%$$

### Electrochemical characterization of Co-PPy-TsOH/C catalysts

Electrochemical performance evaluation of the Co-PPy-TsOH/C catalysts was performed at room temperature of about 25°C with a standard three-electrode system. A Pt wire was used as the counter electrode, while a saturated calomel electrode (SCE) was used as the reference electrode and a catalyst-covered glassy carbon disk with a diameter of 4 mm as the working electrode. A 0.5 M H_2_SO_4_ aqueous solution was used as the supporting electrolyte. The ink-type working electrode was fabricated with the following steps: a catalyst ink was prepared using 6 mg catalyst and 50 μL Nafion® solution (5 wt%, DuPont, Wilmington, DE, USA) in 1 ml double-distilled water. After sonicating for 30 min, 10 μl of the ink was deposited onto the glassy carbon disk to completely cover the surface with a thin film and then air-dried. The catalyst was electrochemically activated by repeatedly scanning the potential in a range from 0.8 to −0.2 V (vs*.* SCE) at a rate of 50 mV · s^−1^ in an oxygen-saturated H_2_SO_4_ solution until stable voltammograms were achieved. Then, the cyclic voltammogram (*CV*) curve was recorded, in oxygen-saturated 0.5 M H_2_SO_4_ solution, in the same potential range at a scan rate of 5 mV · s^−1^ controlled by an electrochemical station (CHI instrument, Austin, TX, USA). The rotating disk electrode (RDE) measurement of the catalysts after activation was conducted by scanning the electrode potential from 0.8 to −0.2 V (vs*.* SCE) at a rate of 5 mV · s^−1^ and with an electrode rotating rate of 900 rpm in argon and oxygen-saturated 0.5 M H_2_SO_4_ solution, respectively. The rotating ring-disk electrode (RRDE) measurement was conducted with the same three-electrode system controlled by a CHI 750 bipotentiostat (CHI instrument, USA) along with a model 636 RRDE system (Pine Instrument, Grove City, PA, USA). A RRDE was employed as the working electrode, while the counter electrode, the reference electrode, and the electrolyte were the same as described above. During the working electrode fabrication, 20 μl of the catalyst ink was spread onto the surface of the disk only. The polarization curves were measured in argon and oxygen-saturated 0.5 M H_2_SO_4_ solution, respectively, at a potential scanning rate of 5 mV · s^−1^ from 0.8 to −0.2 V (vs*.* SCE), electrode rotating rate of 900 rpm and ring potential of 1.0 V (vs*.* SCE).

In the following contents, all the potentials reported are quoted to normal hydrogen electrode (NHE) except specially stated.

### Physicochemical characterization of Co-PPy-TsOH/C catalysts

Crystal/phase structure of the Co-PPy-TsOH/C catalysts were identified by a Rigaku D/MAX-2200/PC XRD instrument (Shibuya-ku, Japan) using Cu *K*α radiation (*λ* = 1.546 Å) at a tube current of 30 mA and a tube potential of 40 kV. The scanned two-theta range was from 20° to 80° at a rate of 6° · min^−1^ with a step size of 0.02°.

Microstructure of the Co-PPy-TsOH/C catalysts was recorded on a JEOL JEM-2100 TEM instrument (Akishima-shi, Japan) operated at 200 kV. After ultrasonic dispersion in ethanol, a drop of the sample was dispersed on a Cu grid for analysis under different magnifications.

Raman spectra of the Co-PPy-TsOH/C catalysts were captured on a UV–vis Raman System 1000 equipped with a charge-coupled device (CCD) detector (Renishaw, Wotton-under-Edge, UK). A CCD camera system with monitor was used to select the location on the sample from which the Raman spectra were taken. Each Raman spectrum was calibrated by an external pen-ray Ne-lamp.

Chemical state of nitrogen in the Co-PPy-TsOH/C catalysts was acquired on a PHI Quantum 2000 XPS instrument (Chanhassen, MN, USA) using Al *K*α radiation with a power of 250 W and pass energy of 14 eV. The data analysis was conducted by AugerScan3.21 software and the peak fitting was carried out with XPS Peak 4.1 software.

Cobalt content in the Co-PPy-TsOH/C catalysts was detected by a Thermal iCAP 6000 Radial ICP spectrometer (Thermo Fisher Scientific, Waltham, MA, USA). By soaking the catalyst samples in aqua regia, cobalt ions can be dissolved in the solution. The content of cobalt in the catalysts can then be determined by measuring the concentration of Co ions in the solution. Contents of non-metallic elements, including N, C, S, and H, in the Co-PPy-TsOH/C catalysts were determined by EA with a PerkinElmer PE 2400 II CHNS/O analyzer (Waltham, MA, USA). To ensure the reliability of the results, each sample was measured twice and the average was recorded as the elemental content. The residual other than Co, N, C, S, and H was calculated to be the oxygen content.

EXAFS analysis of the Co-PPy-TsOH/C catalysts was performed at beamline BL14W1 of the Shanghai Synchrotron Radiation Facility (SSRF). Si (111) double-crystal monochromator was used to select the energy. X-ray absorption data were collected at room temperature in the transmission mode. Gas ion chamber detectors were used. The specimens were prepared by brushing the powders over an adhesive tape and folding it several times for uniformity. Some samples were also made as pellets. Data processing and analysis were done with IFEFFIT software.

## Results and discussion

**Figure 1:**
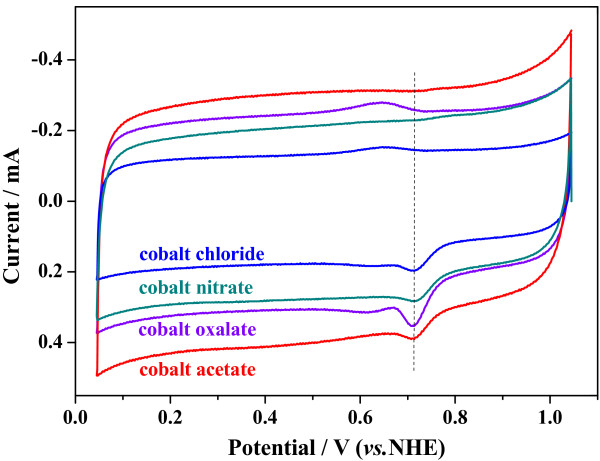
***CV*** **curves of Co-PPy-TsOH/C catalysts prepared from various cobalt precursors in oxygen-saturated 0.5 M H**_**2**_**SO**_**4 **_**solution.**

**Figure 2:**
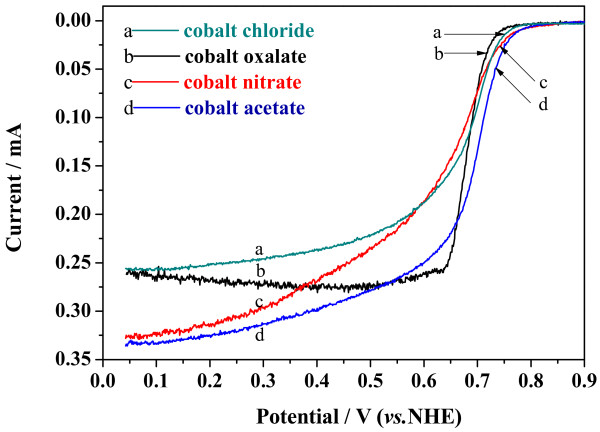
RDE polarization curves of Co-PPy-TsOH/C catalysts prepared from various cobalt precursors.

*CV* curves of the Co-PPy-TsOH/C catalysts prepared from various cobalt precursors in oxygen saturated 0.5 M H_2_SO_4_ are illustrated in Figure 1. No apparent difference in the ORR peak potential, which is traditionally used as a criterion to evaluate the catalytic performance, can be observed; all the peak potentials are about 0.71 V. In the background-corrected RDE polarization curves (Figure 2) which reflect the ORR onset potential and the faradic current, however, obvious difference is demonstrated. The ORR onset potential of the catalysts follows the order, with respect to the cobalt precursor, that cobalt acetate > cobalt nitrate > cobalt chloride > cobalt oxalate. And the faradic current follows the same order in the potential range larger than 0.7 V, where the electrode reaction is mainly controlled by electrochemical process. Therefore, it could be figured out that the cobalt precursors have essential influence on the ORR activity of Co-PPy-TsOH/C catalysts, the catalyst prepared from cobalt acetate has the highest activity, and the catalytic activity follows the order, with respect to the cobalt precursor, that cobalt acetate > cobalt nitrate > cobalt chloride > cobalt oxalate.

**Figure 3:**
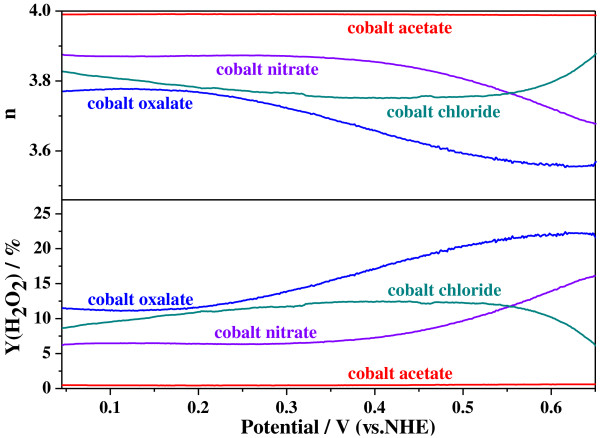
**Calculated values of *n *** **and *Y*** **(H**_**2**_**O**_**2**_**) during ORR catalyzed by Co-PPy-TsOH/C catalysts prepared from various cobalt precursors.**

In order to further explore the ORR mechanism on the Co-PPy-TsOH/C catalysts prepared from diverse cobalt precursors, RRDE technique was employed to detect the formed hydrogen peroxide and the selectivity to four-electron-transfer ORR. In general, the yield of hydrogen peroxide, *Y*(H_2_O_2_), and the number of transferred-electron (*n*) can be estimated from the RRDE experimental data with the following equations [24,25]:

(2) $$n=\frac{4I}{I_{D}+I_{R}/N}$$

(3) $$Y\left(H_{2}O_{2}\right)=\frac{4-n}{2}$$

where *I*_D_ is the disk current, *I*_R_ is the ring current, and *N* is the collection efficiency of RRDE. In the present work, the value of *N* is 0.22. During the actual calculation, the valid potential range is usually chosen from 0.1 to 0.6 V since the values of *I*_D_ and *I*_R_ are too small when the potential is larger than 0.6 V leading to a huge error [26]. The calculated values of *Y*(H_2_O_2_) and *n* from the RRDE data are presented in Figure 3 as function of the potential. It is revealed that the hydrogen peroxide yield and the transferred-electron number are strongly potential dependent, the former decreases with decrease in the disk potential, while the later decreases with increase in the disk potential. However, the relativity remains the same in the whole potential range lower than 0.55 V, the trend for *n*, with respect to cobalt precursor, is cobalt acetate > cobalt nitrate > cobalt chloride > cobalt oxalate, while that for *Y*(H_2_O_2_) is just the opposite. This discloses different ORR mechanism by the Co-PPy-TsOH/C catalysts prepared with different cobalt precursors. The ORR catalyzed by the catalyst with cobalt acetate as precursor proceeds radically through four-electron-transfer reaction, since its calculated electron-transfer number reaches 3.99 in the whole studied potential range. However, it could be obviously acquired that the electron-transfer number of the catalysts prepared from the other salts are evidently lower than 4, indicating that the catalyzed ORR progresses through both two-electron-transfer reduction and four-electron-transfer reduction, while the latter is dominant. Therefore, it could be concluded that cobalt precursors have significant influence on ORR mechanism of the synthesized catalyst Co-PPy-TsOH/C, the selectivity to four-electron-transfer reaction to produce H_2_O follows the order that cobalt acetate > cobalt nitrate > cobalt chloride > cobalt oxalate. This agrees well with the order of catalytic activities discussed above.

Hereto, it could be summarized with the electrochemical study of CV, RDE, and RRDE experiments that the cobalt precursor for the Co-PPy-TsOH/C catalysts significantly affects the ORR activity as well as the mechanism. The electrochemical performance, including both the ORR activity and four-electron-transfer reaction selectivity, of the Co-PPy-TsOH/C catalysts decrease in the order that cobalt acetate > cobalt nitrate > cobalt chloride > cobalt oxalate.

**Figure 4:**
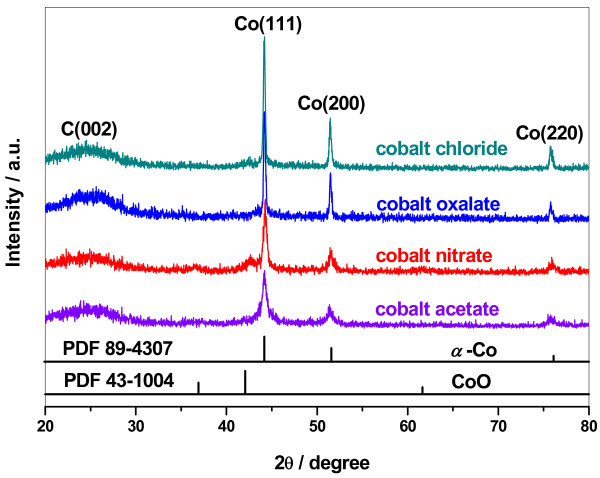
XRD patterns of Co-PPy-TsOH/C catalysts prepared from various cobalt precursors.

To understand the effects of cobalt precursor on electrochemical performance of the corresponding Co-PPy-TsOH/C catalysts, many physicochemical techniques have been employed in this work. Figure 4 presents XRD patterns of the Co-PPy-TsOH/C catalysts prepared from various precursors, the standard data for CoO and *α*-Co are also shown for comparison. Four apparent characteristic peaks can be clearly observed at 2*θ* of 24.5°, 44.2°, 51.5°, and 75.8° in all of the synthesized catalysts, which could be assigned to C(002), Co(111), Co(200), and Co(220) plane. This suggests that cobalt in the Co-PPy-TsOH/C catalysts exists mainly as metallic *α*-Co with face-centered cubic (*fcc*) structure. The Co(111) and Co(200) peaks become sharper and sharper with the order of cobalt acetate, cobalt nitrate, cobalt chloride and cobalt oxalate, implying a growth in the crystallite size of metallic cobalt. Generally, an average crystallite size, *d,* can be estimated with the Shcherrer equation [27,28]:

(4) $$d=\frac{0.89\lambda }{B\;\cos\theta }$$

where *λ* is the wavelength of incident X-ray, *θ* is the incident angle of X-ray for a specific mirror, and *B* is the half-peak width. In order to avoid the interference of CoO on the Co(111) plane, the Co(200) plane was adopted in this study to calculate the crystallite size of metallic cobalt. The calculated specific values are listed in Table 1. It can be inferred that the relativity of the crystallite size of metallic cobalt in the catalysts is exactly opposite to the trend of ORR performance. In addition, two weak diffraction peaks observed at 2*θ* of 36.5° and 42.2° indicate the co-existence of a very small amount of CoO (PDF 43–1004) in the catalysts. Therefore, it could be figured out that the crystallite size of metallic cobalt in the catalysts has essential influence on the catalytic performance towards ORR, the smaller the crystallite size, the better the performance. A small-amount co-existence of CoO in the catalysts does not have an adverse effect on the performance. But on the contrary, it is probably that the synergetic effect between metallic cobalt and the oxide may effectively enhance the catalytic performance as presented by previous researches [29,30].

**Table 1:** Crystallite size of metallic cobalt in Co-PPy-TsOH/C catalysts prepared from various cobalt precursors

**Cobalt precursor**	**Crystallite size of metallic co/nm**
Cobalt acetate	0.4253
Cobalt nitrate	0.4947
Cobalt oxalate	0.6432
Cobalt chloride	0.6099

**Figure 5:**
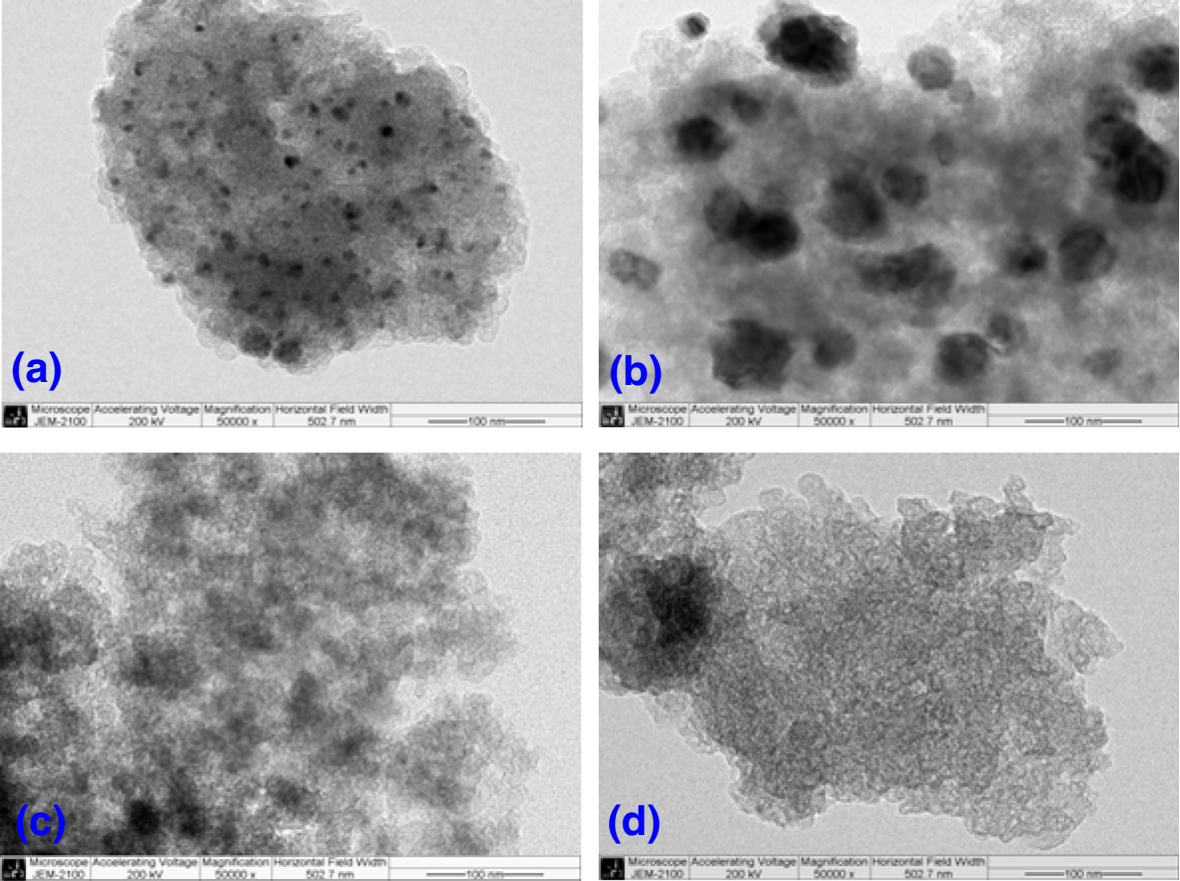
**TEM images of Co-PPy-TsOH/C catalysts prepared from various cobalt precursors. (a)** Cobalt acetate; **(b)** cobalt nitrate; **(c)** cobalt oxalate; **(d)** cobalt chloride.

Figure 5 displays TEM images of the Co-PPy-TsOH/C catalysts prepared from various precursors. Small and uniformly distributed metallic cobalt particles can be clearly seen in the catalyst with cobalt acetate as precursor. Yet, when cobalt nitrate is used as the precursor, serious agglomeration of the catalyst particles can be found, the particle size even reaches as large as 50 nm. However, the morphology of the catalysts prepared with cobalt chloride and cobalt oxalate is much different, and no clear metallic cobalt particles could be observed. At present, we cannot well understand this phenomenon, but we presume that it is because of the serious reunion of the metallic cobalt particles since XRD results have revealed much larger crystallite size of metallic cobalt in these catalysts than in those prepared with cobalt acetate and cobalt nitrate as precursors. These results disclose that small Co particles and the uniform dispersion are beneficial for obtaining a high-performance Co-PPy-TsOH/C catalyst towards ORR, while large cobalt particles and the agglomeration deteriorate the catalytic performance.

**Figure 6:**
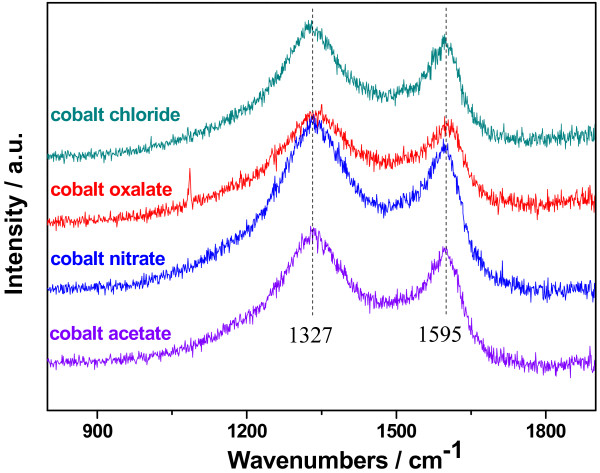
Raman spectra of Co-PPy-TsOH/C catalysts prepared from various cobalt precursors.

Figure 6 demonstrates Raman spectra of the Co-PPy-TsOH/C catalysts prepared from various cobalt precursors. As in our previous work [10,23], the characteristic peaks generally observed in the wavenumber range from about 900 to 1,150 cm^−1^ for PPy and 1,370 cm^−1^ for antisymmetric in-ring C-N stretching [31,32] disappeared in all the obtained catalysts, while only two peaks representing the disorder-induced band (*D* band, 1,327 cm^−1^) and the graphite band (*G* band, 1,595 cm^−1^) for carbon can be found, indicating the decomposition of PPy and insertion of nitrogen into the carbon layers during high-temperature pyrolysis. Usually, the graphitization degree of carbon materials can be estimated with the ratio of the *G* band and *D* band intensities (*I*_*G*_/*I*_*D*_), the higher the ratio, the larger the graphitization degree [33]. For the studied catalysts in the present work, the values of *I*_*D*_ and *I*_*G*_ extracted from Figure 6 along with the calculated values of *I*_*G*_/*I*_*D*_ are listed in Table 2. An inverse order of the graphitization degree is exhibited to that of catalytic performance, resulting from the reconfiguration of nitrogen-impregnated graphitic carbon. So, it could be summarized that the graphitization degree of carbon in the Co-PPy-TsOH/C catalysts plays significant role on the catalytic performance towards ORR, the lower the graphitization degree, the better the catalytic performance. It is worthwhile to note that this relationship between the graphitization degree of carbon and the catalytic properties of Co-PPy-TsOH/C catalysts is just opposite to that drawn by Choi et al. [34] for nitrogen-containing carbon-based catalyst for ORR. We cannot, at present, well understand this discrepancy, but we believe one of the probable reasons is the different preparation of the catalysts and the different carbon and nitrogen sources used, resulting in different microstructure. In Choi et al.'s research [34], the catalysts were prepared through pyrolysis of polymer, dicyandiamide, with/without metal precursors where the polymer was used as the source for both carbon and nitrogen. In the present work, however, the catalysts were obtained by pyrolyzing TsOH-doped PPy-modified carbon black with cobalt ion impregnated on the surface, the carbon in the obtained catalyst is mainly from BP2000 in addition to a small part from PPy and the nitrogen is mainly from PPy.

**Table 2:** ***D*-band and *G *-band intensities of carbon in Co-PPy-TsOH/C catalysts prepared from various cobalt precursors and calculated graphitization degree**

**Cobalt precursor**	***D*-band intensity (*I***_***D***_**/a.u.)**	***G*-band intensity (*I***_***G***_**/a.u.)**	**Graphitization degree (*I***_***G***_**/*I***_***D***_**)**
Cobalt acetate	2,122	1,768	0.833
Cobalt nitrate	2,678	2,377	0.887
Cobalt oxalate	1,633	1,493	0.914
Cobalt chloride	2,158	1,942	0.900

**Figure 7:**
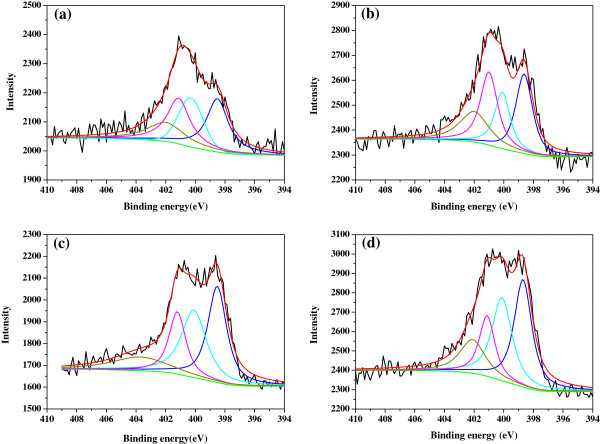
**XPS spectra for N1s core-level peaks in Co-PPy-TsOH/C catalysts prepared from various cobalt precursors. (a)** Cobalt acetate; **(b)** cobalt nitrate; **(c)** cobalt oxalate; **(d)** cobalt chloride.

It has been reported [16,35,36] that N1s peaks in XPS spectra can be decomposed into four types according to the binding energy: (1) pyridinic-N (398.0 to 399.5 eV, a nitrogen atom bonded to two carbon atoms on the edge of a graphene layer, contributing to the *π* system with one *p* electron); (2) pyrrolic-N (400.1 to 400.9 eV, a nitrogen atom bonded to two carbon atoms and one hydrogen atom on the edge of a graphene layer, contributing to the *π* system with two *p* electrons); (3) graphitic-N (401 to 402 eV, highly coordinated nitrogen atoms such as N atoms bound to three carbon atoms in different locations of a graphene layer); and (4) oxidized-N (402 to 410 eV). The function of these types of nitrogen towards ORR in transition metal-based nitrogen-containing catalysts has also been discussed in the literatures. For example, pyridinic-N has been considered by many researchers [37] to be responsible for the ORR catalytic performance, and Faubert et al.'s investigation [17] revealed that pyridinic-N is involved in the composition of the catalytic site for ORR in Fe-based catalysts obtained at high pyrolysis temperatures, but other types of nitrogen including pyrrolic-N do not seem to be involved. However, the study on heat-treated Fe-based and Co-based nitrogen-containing catalysts by Faubert et al. [38] and Yang et al. [39] showed that decrease in pyridinic-N and increase in pyrrolic-N lead to enhanced ORR catalytic performance. Besides, the importance of graphitic-N to enhancing the ORR catalytic performance has been emphasized by Niwa et al. [40] and Nagaiah et al. [41]. The reason for the huge discrepancy between these results is unclear, but it is probably, at least in part, resulted from different catalyst synthesis, metal precursor, nitrogen source, and so on. In the present work, it could be found in Figure 7 which shows the XPS spectra for N1s in the Co-PPy-TsOH/C catalysts prepared with various cobalt precursors that the chemical state of nitrogen in the catalysts is greatly dependent on the used cobalt precursor for catalyst synthesis, and all the four types of nitrogen could be observed in the studied catalysts, suggesting that the nitrogen atoms have successfully incorporated into the carbon structures and replaced carbon atoms located at the edges and inside of the graphitic carbon layer. Outwardly, the N1 spectra of the catalysts synthesized with cobalt acetate and cobalt nitrate are apparently different from that with cobalt oxalate and cobalt chloride. The peak at about 401 eV is obviously higher than that at about 398 eV for the former, while the height of these peaks is almost the same for the latter. The spectra in Figure 7 have been deconvoluted into various types of nitrogen as shown and the specific concentration of each state of nitrogen is listed in Table 3. The nitrogen distribution in the studied catalysts can be classified into two groups. Similar results have been obtained in the catalysts prepared from cobalt acetate and cobalt nitrate, and closely similar distributions have been exhibited in the catalysts synthesized from cobalt oxalate and cobalt chloride. This is probably because of the fact that the reconfiguration of the catalyst, especially the decomposition of PPy and the insertion of nitrogen into carbon, during high-temperature pyrolysis could be interfered by the transforming process of cobalt ion in the used precursor into metallic cobalt. When cobalt acetate and cobalt nitrate are used, they thermally decompose under inert atmosphere into cobalt oxide and then metallic cobalt [42-45]. When cobalt oxalate is employed, however, it thermally decomposes into metallic cobalt directly [46-48], and the cobalt ion in cobalt chloride is reduced by carbon directly into metallic cobalt [49,50]. Thus, different states and the corresponding content of nitrogen in the final catalysts have been achieved. As to the correlation between the ORR performance of the catalysts and the concentration of each type of nitrogen in the catalysts, neither positive nor negative trend could be found. Therefore, it is difficult at present to expatiate the specific contribution of each type of nitrogen to the ORR catalytic performance of the Co-PPy-TsOH/C catalysts, maybe synergistic effects exist among them.

**Table 3:** Surface atomic concentration of different types of nitrogen in Co-PPy-TsOH/C catalysts prepared from various cobalt precursors

**Cobalt precursor**	**Pyridinic-N**	**Pyrrolic-N**	**Graphitic-N**	**Oxidized-N**
Cobalt acetate	0.308	0.225	0.279	0.188
Cobalt nitrate	0.297	0.204	0.293	0.207
Cobalt oxalate	0.345	0.305	0.197	0.153
Cobalt chloride	0.355	0.311	0.175	0.159

**Figure 8:**
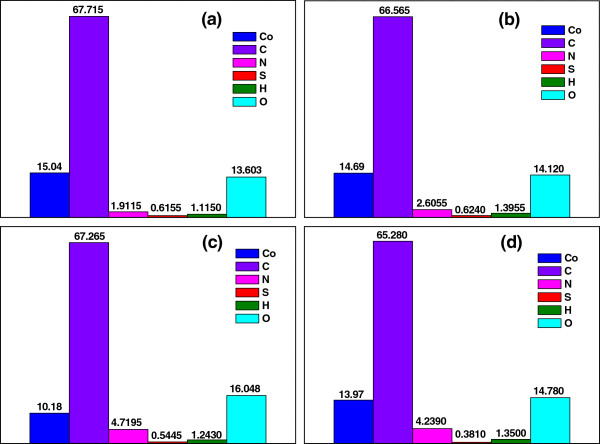
**Elemental contents in Co-PPy-TsOH/C catalysts prepared from various cobalt precursors. (a)** cobalt acetate; **(b)** cobalt nitrate; **(c)** cobalt oxalate; **(d)** cobalt chloride.

Figure 8 exhibits content of diverse elements in the Co-PPy-TsOH/C catalysts prepared with various precursors. Comparable carbon contents have been achieved in the studied catalysts. However, the content of other elements differs greatly from each other. Cobalt content in the catalysts prepared with cobalt acetate, cobalt nitrate, and cobalt chloride is obviously higher than the designed value of 10.55%, while that in the catalyst from cobalt oxalate is slightly lower than 10.55%. This is probably resulted from different removal of various elements such as N, C, S, H, O, and perhaps Co during the high-temperature pyrolysis. Similarly, a different content of N, S, H, and O has been obtained in the catalysts prepared with various cobalt precursors. It can be acquired that Co content in the catalysts follows the order that cobalt acetate > cobalt nitrate > cobalt chloride > cobalt oxalate, matching well with the order of catalytic performance of the catalysts, while the order of nitrogen content is just the opposite. These results strongly disagree with the research in literatures [51-55] on transition metal-based nitrogen-containing catalysts towards ORR. They showed that there is an optimal metal content in the catalyst for obtaining best ORR performance but not larger metal content leading to better performance [51,52], and the more the nitrogen in the catalyst, the higher the catalytic performance [53-55]. For the other elements of C, S, H, and O, a direct relationship between their contents and the catalytic performance could not be figured out. Therefore, it is difficult for us at present to explain the effects of each element and its content in this series of catalysts on the catalytic performance. As discussed above with the N1s XPS spectra, it is probable that the used cobalt precursors and their decomposition/reduction interfere with the pyrolysis process leading to different state of each element in the obtained catalysts and correspondingly different performance. On the other hand, we believe that synergistic effects between the existing elements/states/contents are not negligible and maybe they play very important role on the catalytic performance. More detailed work should be done in the future to find a solid relationship between the elemental contents and the catalytic performance of the Co-PPy-TsOH/C catalysts towards ORR.

**Figure 9:**
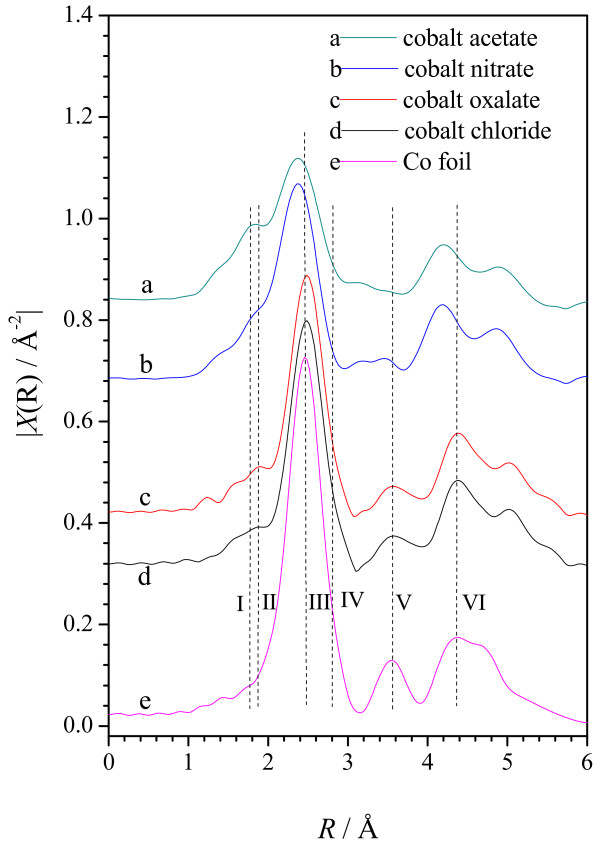
**Fourier-transformed *k***^**3**^**-weighted EXAFS functions at Co K-edge for Co foil and Co-PPy-TsOH/C catalysts prepared with various cobalt precursors**.

Figure 9 demonstrates the Fourier transformed *k*^3^-weighted EXAFS functions at the Co *K*-edge for the Co-PPy-TsOH/C catalysts prepared with various cobalt precursors, the data for Co foil is also presented for comparison. Herein, the labeled peaks could be assigned to Co-N bond (I), Co-O bond (II and IV), the first neighbor shell of Co-Co bond (III), the second neighbor shell of Co-Co bond (V) and the third neighbor shell of Co-Co bond (VI) [56,57]. Obviously, cobalt in the prepared Co-PPy-TsOH/C catalysts exists mainly as metallic cobalt, while only very small amounts of Co-N and/or Co-O structure could be found. This agrees well with the results of the XRD analysis. The peaks representing Co-Co bond in the catalysts from cobalt oxalate and cobalt chloride match well with that of Co foil with slight positive shift of the first and third neighbor shells. For the catalysts from cobalt acetate and cobalt nitrate; however, all the Co-Co neighbor shells shift obviously to negative distance. These results imply that the crystallite size of metallic cobalt in the catalysts prepared from cobalt oxalate and cobalt chloride is obviously larger than that in the other two catalysts, agreeing well with the calculated results from the XRD data. The Co-N structure can be evidently detected in the catalysts synthesized from cobalt acetate, while that in the other catalysts are negligible. Therefore, the EXAFS results suggest that the Co-N bond/structure is not necessary to forming a catalytic active site toward ORR in Co-PPy-TsOH/C catalysts, while the metallic cobalt plays an important role in forming the active site. Smaller Co-Co bond distances/crystallite size is beneficial for enhancing the ORR performance, agreeing well with the results of Yuasa et al. [21]. In their research on Co-PPy/C catalysts, synthesized with electrochemically polymerized PPy, they found that heat-treatment shortens the distances of Co-Co bond leading to better catalytic performance towards ORR.

## Conclusions

Effects of cobalt precursors on electrochemical performance of Co-PPy-TsOH/C as catalyst towards ORR have been comparatively studied, and the results have been analyzed with diverse physiochemical techniques. The following conclusions could be drawn from this research: (1) cobalt precursors affect both the catalytic activity of the Co-PPy-TsOH/C catalysts and the corresponding ORR mechanism; (2) the electrochemical performance, including both the ORR catalytic activity and the selectivity to four-electron-transfer reaction, of the Co-PPy-TsOH/C catalysts follows the order with respect to the used cobalt precursor that cobalt acetate > cobalt nitrate > cobalt chloride > cobalt oxalate; (3) the synthesis process, especially the high-temperature pyrolysis, of the catalyst could be interfered by the used cobalt precursors, resulting in different microstructure, morphology, elemental state as well as the ORR performance; (4) lower graphitization degree of carbon and smaller crystallite/particle size of metallic cobalt and the uniform distribution in Co-PPy-TsOH/C catalysts lead to better ORR performance; (5) metallic cobalt is a main component forming the ORR active site in the Co-PPy-TsOH/C catalysts, but some other elements such as nitrogen is probably also involved; and (6) Co-N bond/structure is not necessary to forming a catalytic active site toward ORR in Co-PPy-TsOH/C catalysts, and a small-amount coexistence of CoO in the catalysts does not have an adverse effect on the electrochemical performance.

## Competing interests

The authors declare that they have no competing interests.

## Authors' contributions

The manuscript was written through the contributions of all authors, and all the authors have read and approved the final manuscript.
